# Classification based on event in survival machine learning analysis of cardiovascular disease cohort

**DOI:** 10.1186/s12872-023-03328-2

**Published:** 2023-06-20

**Authors:** Shokh Mukhtar Ahmad, Nawzad Muhammed Ahmed

**Affiliations:** 1 Department of Statistics and Informatics, College of Administration and Economics, Sulaymaniyah University, Sulaymaniyah, Kurdistan Iraq; 2 Department of Medical Laboratory, Komar University of Science and Technology Science, Sulaymaniyah, Kurdistan Iraq

**Keywords:** Survival analysis, Machine learning, Logistic regression, SVM, Tree descent, Random forest.

## Abstract

The aim of this study is to assess the effectiveness of supervised learning classification models in predicting patient outcomes in a survival analysis problem involving cardiovascular patients with a significant cured fraction. The sample comprised 919 patients (365 females and 554 males) who were referred to Sulaymaniyah Cardiac Hospital and followed up for a maximum of 650 days between 2021 and 2023. During the research period, 162 patients (17.6%) died, and the cure fraction in this cohort was confirmed using the Mahler and Zhu test (P < 0.01). To determine the best patient status prediction procedure, several machine learning classifications were applied. The patients were classified into alive and dead using various machine learning algorithms, with almost similar results based on several indicators. However, random forest was identified as the best method in most indicators, with an Area under ROC of 0.934. The only weakness of this method was its relatively poor performance in correctly diagnosing deceased patients, whereas SVM with FP Rate of 0.263 performed better in this regard. Logistic and simple regression also showed better performance than other methods, with an Area under ROC of 0.911 and 0.909 respectively.

**Supplementary Information:**

The online version contains supplementary material available at 10.1186/s12872-023-03328-2.

## Background

Heart disease, also known as cardiovascular disease (CVD), is one of the leading causes of death worldwide. According to a report published by the World Health Organization in 2018, approximately 17.9 million people die annually due to this disease. However, Middle Eastern countries are experiencing a much worse situation than other parts of the world. The latest WHO report states that about 19% of all deaths in Iraq are due to coronary heart disease, ranking Iraq 20th in age-adjusted Death Rate at 230.27 per 100,000 people [1].

In recent years, the treatment of many diseases, especially heart disease, has significantly improved. As a result, the number of patients who survive has increased. However, this increase in cured patients who are censored from the data frame requires new methods of survival analysis [2].

Survival analysis is a statistical method used to analyze the time until an event of interest occurs, such as death or disease progression. In recent years, machine learning techniques have been increasingly applied to survival analysis problems in healthcare.

The purpose of the study is to compare and assess the effectiveness of supervised learning classification models in predicting patient outcomes in a survival analysis problem involving cardiovascular patients with a significant cure fraction. The study aims to identify the most effective machine learning algorithm for predicting patient outcomes and to evaluate the performance of different classification indices.

The study uses a cohort of cardiovascular disease patients with a significant cure fraction. The dataset includes demographic information, medical history, laboratory test results, and clinical outcomes. The machine learning methods used in this study include Support Vector Machine (SVM), Logistic Regression, Random tree, random forest, C4.5 algorithm, and compression indices include ROC area and other classification indices.

The findings of this study can help healthcare professionals predict patient outcomes more accurately and improve patient care. The results can also inform future research on machine learning applications in survival analysis problems.

This study builds upon previous research that has demonstrated the potential of machine learning techniques for predicting patient outcomes in survival analysis problems involving CVD patients. By comparing the effectiveness of different supervised learning classification models, this study aims to provide insights into which models are most effective for predicting patient outcomes in this context [3–5].

## Methods

Survival analysis includes many methods to model and predict the probability of survival up to a certain time t, $\text{P}\left(\text{T}>\text{t}\right)$ where $\text{T}$ is the survival time random variable;

1 $$\text{S}\left(\text{t}\right) = \text{P} (\text{T} > \text{t}) = {\int }_{\text{t}}^{{\infty }}\text{f} \left(\text{u}\right)\text{d}\text{u} = 1 - \text{F} \left(\text{t}\right),$$

To better estimate this probability, covariates variables such as $({\text{x}}_{1}, {\text{x}}_{2}, \dots {\text{x}}_{\text{k}})$ are used in statistical models.

The most widely used survival analysis model is the Cox model. This pseudo-regression models and predicts the mentioned probability with the following function:

2 $$\begin{array}{l}\lambda \left( {\rm{t}} \right) = {\lambda _0}\left( {\rm{t}} \right){\rm{exp}}\left( {{\beta _1}{{\rm{x}}_1} + {\beta _2}{{\rm{x}}_2} + \ldots + {\beta _{\rm{k}}}{{\rm{x}}_{\rm{k}}}} \right)\\= {\lambda _0}\left( {\rm{t}} \right){\rm{exp}}(\sum\limits_{{\rm{j}} = 1}^{\rm{k}} {{{\rm{x}}_{{\rm{ij}}}}} {\beta _{\rm{j}}})\end{array}$$

In Eq. (2) the response variable is the hazard function ʎ(t), which assesses the probability that the event of interest (in this case, death) occurred at time of t. The equation models this hazard as an exponential function of an arbitrary baseline hazard ʎ_0_(t) when all covariates are null, and β is the regression coefficient of the covariates, $({\text{x}}_{1}, {\text{x}}_{2}, \dots {\text{x}}_{\text{k}})$ [6].

On the other hand, hazard and survival function are related, so that:

3 $$\lambda \left( {\rm{t}} \right) = - \frac{{{\rm{dlogS}}\left( {\rm{t}} \right)}}{{{\rm{dt}}}} = \frac{{{\rm{f}}\left( {\rm{t}} \right)}}{{{\rm{S}}\left( {\rm{t}} \right)}}$$

In Eq. (2), it can be seen that the logarithm of the hazard function is a multiple regression on multi-dimensional covariates, but the very important difference between this model and regression is due to the data. In the survival analysis, the data frame consists of two groups of patients. One of the groups has experienced the event under study (which is death here), but the second group of patients was still alive at least during the studied time period, that’s why we call them sensors from the right. Therefore, the Cox proportional hazard (CPH) model is a special type of regression considering time-to-event data.

In the CPH model, partial likelihood is maximized for estimation and inference on the parameter β:

4 $$\begin{array}{l}{\rm{L}}\left( \beta \right) = \prod\limits_{\rm{i}} {{{\rm{L}}_{\rm{i}}}} \left( \beta \right) = \prod\limits_{\rm{i}} {\frac{{\lambda ({{\rm{y}}_{\rm{i}}}\left| {{{\rm{x}}_{\rm{i}}})} \right.}}{{\sum\limits_{{{\rm{i}}^\prime }:{{\rm{y}}_{{{\rm{i}}^\prime }}} \ge {{\rm{y}}_{\rm{i}}}} \lambda ({{\rm{y}}_{\rm{i}}}\left| {{{\rm{x}}_{\rm{i}}})} \right.}}} \\= \prod\limits_{\rm{i}} {\frac{{{\rm{exp}}(\sum\limits_{{\rm{j}} = 1}^{\rm{k}} {{{\rm{x}}_{{\rm{ij}}}}} {\beta _{\rm{j}}})}}{{\sum\limits_{{{\rm{i}}^\prime }:{{\rm{y}}_{{{\rm{i}}^\prime }}} \ge {{\rm{y}}_{\rm{i}}}} {\rm{e}} {\rm{xp}}(\sum\limits_{{\rm{j}} = 1}^{\rm{k}} {{{\rm{x}}_{{\rm{ij}}}}} {\beta _{\rm{j}}})}}} \end{array}$$

After estimating the parameters in the CPH model, another important issue is choosing the variables to include in the model. This topic has also been studied in many research studies. In [7], the lasso method for variable selection is proposed, in [8], smoothly clipped absolute deviation is presented, and in [9], an adaptive lasso method is also introduced.

Also [10], in their research, using a new method called “stacking”, they introduced the problem of survival analysis only as a classification problem. They also used several machine learning methods in addition to the Cox model in order to classify the subjects into two classes, alive and dead.

Although the most important issue in survival analysis is the probability of surviving until a particular time, predicting that a person belonging to the category of patients with their unique characteristics will survive or die during a certain time is also a very important issue in survival analysis. For this purpose, in this article, we have compared the results of different binary classification methods.

Since there is a wide range of classification methods, we have selected some of them for this research. Logistic regression is perhaps the most famous statistical method that has been frequently used in survival analysis. Also, machine learning methods such as random decision tree, J48, and random forest, have been considered. In addition to them, the support vector machine (SVM) method is a very interesting method with the lowest risk of assigning subjects to groups, and is also one of the favorite techniques in survival analysis. In section two, a brief introduction of each of these methods has been discussed. In section three, the data used in this research are introduced, and practically each of the five classification methods is applied to them. Their results will be compared and discussed in section five.

## Survival machine learning analysis

In clinical research, we deal often with high dimensional data that contains missing and censored data. Demographic status, physical conditions, and hospital interventions are all covariates that help us predict the patient’s condition during the study period. In addition to classical statistical methods such as regression, machine learning methods have attracted much attention from medical researcher due to their simplicity and sometimes more accurate predictions. Recently, many studies have compared machine learning methods in survival analysis [11, 12].

Machine learning techniques, which are non-parametric and less complex, are good alternatives to statistical methods. Users mostly like these methods because of their simplicity and because the results are often more accurate and close to reality.

The decision tree, as one of them after being introduced by [13], is a very flexible and easy-to-interpret model. Recently due to many research studies, tree-based methods have improved significantly. The random forests technique [14] has become an excellent method in machine learning. Meanwhile, the use of tree-based methods for survival analysis has drawn a lot interest. So much research has focused on tree building and dealing with censoring.

It is very important to remember that the purpose of survival analysis is to predict the survival time of patients in a cohort based on the available data. Although machine learning methods have been successful in achieving this goal in many ways due to the lack of complexities that exist in classical statistical models such as the Cox model, they also have some weaknesses. For example, in SVM survival analysis, predictions for survival time are made by ranking patients according to the probability of death. In other words, its results are obtained in the form of a rank. This issue makes it difficult to compare its results with classic forms of survival analysis such as CPH [15, 16]. Other techniques, such as random forest, have also been used in survival analysis. Random survival forests (RFS) land marking as a nonparametric, machine learning alternative for obtaining dynamic predictions when there are complex or unknown relationships present is introduced. It requires little upfront decision-making, has comparable predictive performance, and has preferable computational speed [17].

Of course, in this paper, several methods of machine learning will be used as binary classification methods in order to determine the survival or death of patients during treatment. This means that the problem of censoring will be just predicted variable. Their results will be compared using classification evaluation indices.

## Results

In this paper, a sample of 919 patients referred to Sulaymaniyah Cardiac Hospital (including 365 females and 554 males) were followed up for a maximum of 650 days in 2021 to 2023. In the sample, 162 patients (17.6%) died during research time. Since the presence of cure fraction in these data was confirmed based on the Mahler and Zhu test (P < 0.01), mixture cure models based on various probability distributions were used [18].

In this section, as a classification problem, two groups of survivors and dead during the follow-up period of the data have been discussed using of some variables. There are three sets of covariates used in this research: demographics, selected blood sample markers, and medical interventions.

Demographics: This set includes four variables: Gender, Age, Job, and Location. These variables are qualitative in nature as they represent categorical data.

Selected blood sample markers: This set includes 11 variables: Glucose, Creatine, urea, WBC (white blood cells), LYM (lymphocytes), MID (mid-range white blood cells), GRA (granulocytes), HGB (hemoglobin), RBC (red blood cells), MCV (mean corpuscular volume), PLT (platelets). These variables are quantitative in nature as they represent numerical data.

Medical interventions: This set includes three variables - Doctor, Coronary angio, Coronary angio, and PCI, CABG. These variables are qualitative in nature as they represent categorical data.

In total there are 19 variables used in this research. The demographics and medical intervention variables are qualitative, while the selected blood sample markers are quantitative. These are presented in Table 1.

**Table 1:** Covariates selected for patient classification

Categories	Covariates
Demographic variables	Gender
	Age
	Job
	Location
Selected blood sample markers	Glucose
	Creatine
	Urea
	WBC
	LYM
	MID
	GRA
	HGB
	RBC
	MCV
	PLT
Medical interventions	Doctor
	Coronary angio
	Coronary angio &PCI
	CABG

The amount of missing data in covariates can have a significant impact on the accuracy and reliability of machine learning classification methods. While there is no universally agreed-upon maximum percentage of missing data, several studies have suggested that missing data rates above 5–10% can lead to biased or inaccurate results [19, 20].It should be noted that in this research, fortunately, out of 19 covariates, only 3 variables, Glucose, Creatine, and Urea had more than 5% missing data.

**Fig. 1:**
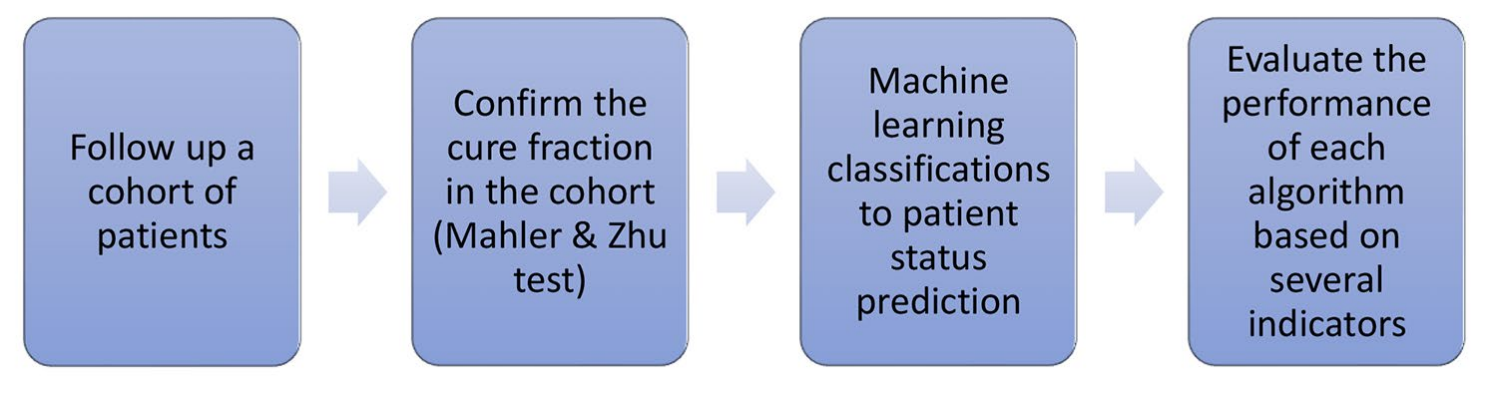
Workflow chart in this research

In Fig. 1 the step-by-step process for completing the task at hand is illustrated clearly.

In this article, Weka software package was used to perform the analysis. Weka splits the data into training and testing data by default. The default setting is a 66% training set and a 34% testing set.

## Classification results

This research aimed to classify patients into two categories using standard machine learning methods without any additional rules. The results of the classification were analyzed using various indices to evaluate the performance of different machine learning methods in classifying the final patient’s status.

Table 2 presents confusion matrices according to classification methods, which were used to obtain the Table 3 indices.

**Table 2:** Confusion matrices according to classification methods

Classification	Random forest		Logistic		Regression		C4.5 tree		Random tree		SVM	
Patient Status\Prediction	Alive	dead	Alive	dead	Alive	dead	Alive	dead	Alive	dead	Alive	dead
Alive	748	9	729	28	739	18	740	17	691	66	742	15
Dead	52	110	51	111	55	107	55	107	67	95	51	111

**Table 3:** Classification indices according to classification methods

	TP Rate	FP Rate	Precision	Recall	F-Measure	MCC	ROC Area	PRC Area
Random forest	0.934	0.267	0.933	0.934	0.929	0.757	0.934	0.961
Logistic	0.914	0.266	0.911	0.914	0.911	0.689	0.911	0.933
Regression	0.921	0.284	0.918	0.921	0.916	0.708	0.909	0.934
C4.5 tree	0.922	0.284	0.919	0.922	0.917	0.712	0.844	0.902
Random tree	0.855	0.356	0.855	0.855	0.855	0.500	0.843	0.878
SVM	0.928	0.263	0.926	0.928	0.925	0.737	0.833	0.885

The results presented in Table 3 indicate that random forest outperformed other methods in all indices except for the FP Rate index. On the other hand, SVM performed well in all indicators, especially the FP rate, but had the lowest area under the ROC. Statistical methods such as logistic and simple regression showed relatively balanced performance across all indicators.

**Fig. 2:**
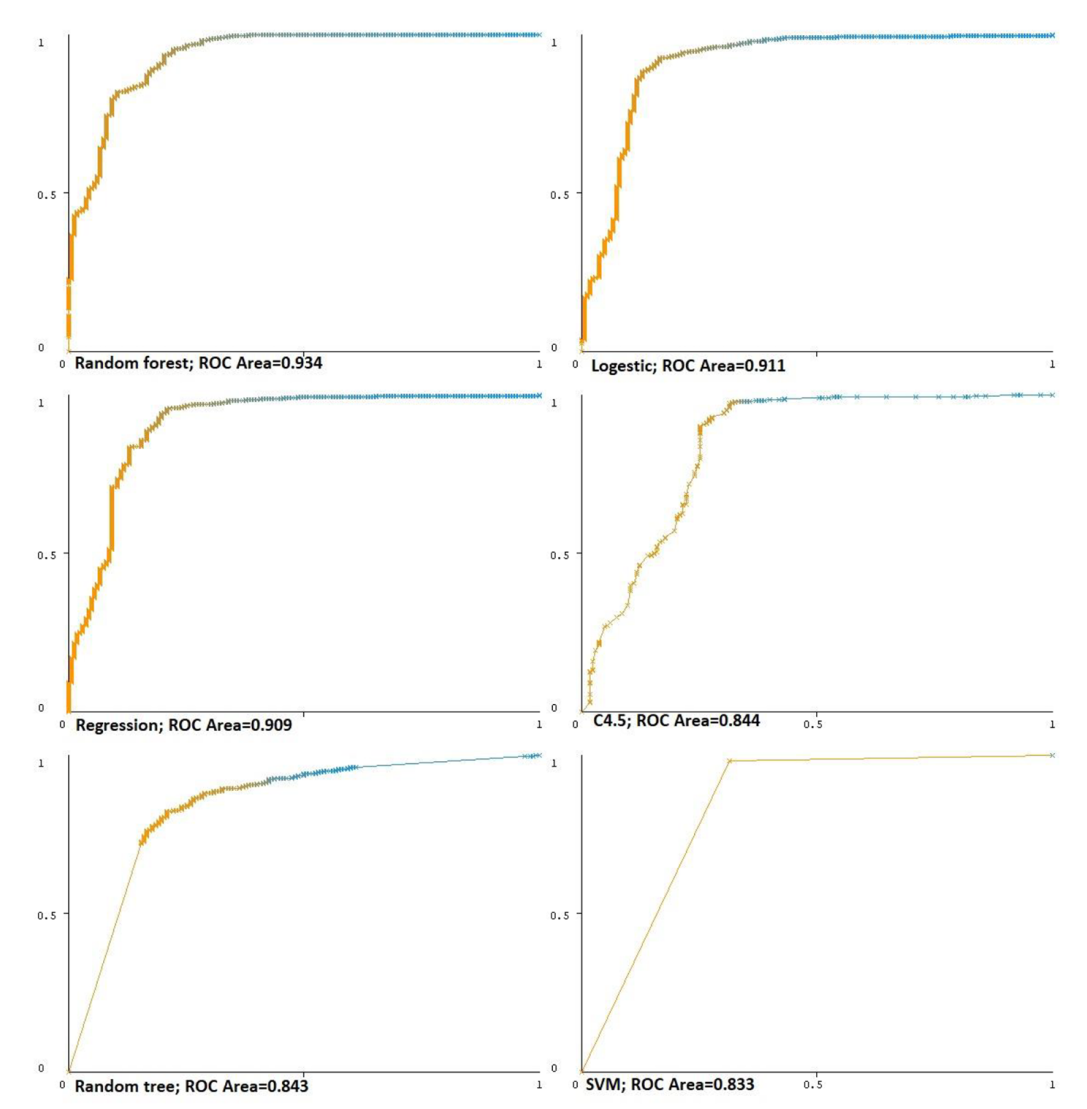
Receiver operating characteristic (ROC) plots according to classification methods

Figure 2 presents Receiver Operating Characteristic (ROC) plots according to classification methods. The area under the ROC curve, which indicates the avoidance of false positive diagnosis and the tendency to correct positive diagnoses, was greater than 0.5 for all selected methods. Random forest showed the greatest avoidance of false positives and the tendency to correctly recognize positives.

This research demonstrates that standard machine learning methods can effectively classify patients into two categories without any additional rules. The results suggest that different machine learning methods have varying strengths and weaknesses in terms of their performance across different indicators.

## Discussion

The present study aimed to evaluate the effectiveness of supervised learning classification models in predicting patient outcomes in a survival analysis problem involving cardiovascular patients with a significant cure fraction. The results of this study demonstrate that machine learning algorithms can be used to accurately predict patient outcomes in a clinical setting.

One of the key advantages of using machine learning algorithms is their ability to analyze large amounts of data quickly and accurately. In this study, the sample size comprised 919 patients, which is a relatively large sample size for a clinical study. The use of machine learning algorithms allowed for the analysis of this large dataset in an efficient and effective manner.

Another advantage of using machine learning algorithms is their ability to identify patterns and relationships within the data that may not be immediately apparent. In this study, several machine learning classifications were applied to classify patients into alive and dead categories. The results showed that random forest was identified as the best method for most indicators, with an Area under the ROC of 0.934. This indicates that random forest was able to accurately predict patient outcomes based on several indicators.

Furthermore, logistic and simple regression also showed better performance than other methods, with an Area under ROC of 0.911 and 0.909 respectively. These findings suggest that these methods could also be used effectively to predict patient outcomes.

However, it should be noted that there were some limitations to this study. One weakness of the random forest method was its relatively poor performance in correctly diagnosing deceased patients, whereas SVM with a FP Rate of 0.263 performed better in this regard.

In conclusion, the present study demonstrates that supervised learning classification models can be used effectively to predict patient outcomes in a clinical setting involving cardiovascular patients with a significant cure fraction. The use of machine learning algorithms allows for efficient and accurate analysis of large datasets and can identify patterns and relationships within the data that may not be immediately apparent using traditional statistical methods.

## Conclusions

Although heart disease is one of the most widespread diseases and causes of death in the world, especially in the Middle East, the improvement of hospital and treatment services has led to the recovery of a significant part of these patients and their return to normal life. In a time-to-event problem, in order to predict the survival probability of each patient until a certain time in such conditions, it requires more complete models than Cox models, which are called Cure models. On the other hand, machine learning has caught the attention of researchers in this field as a simpler method with reality results. The output of survival machine learning is based on the rank of patient’s death. In this research, the survival problem is reduced to just prediction during the follow-up so that the results of several machine learning methods can be checked in such a situation.

In the results, we saw that random forest performed better based on all criteria except false positive rate. The reason for this is the high risk of this method in the problem of survival detection, which has led to misdiagnosis of some dead patients as cured. Contrary to that, since SVM is a minimum risk classification method in determining separation vectors, it has acted more conservatively. Although this conservatism in detecting survival has the lowest false positive rate among other methods, but due to the problem with presence of a significant cured fraction of patients has caused this method to have the worst performance in the important indicator of the area under the ROC. On the other hand, the presence of many variables related to death in medical issues has caused classical statistical methods such as logistic and simple regression to be in relatively ideal conditions in all indicators after random forest. In general, since the ROC curve indicates the avoidance of wrong diagnosis and the tendency towards the correct diagnosis in patients’ lives, it was taken into consideration. Based on this criterion, random forest performed best and SVM performed worst. Therefore, conservative methods such as SVM are not recommended in problems like this, which has a significant survival expectation.

## Electronic supplementary material

Below is the link to the electronic supplementary material.

Additional File 1: Data set used in this research.

Additional File 2: All the Syntax used for running the application part.

## Data Availability

The datasets of the current study are available from the corresponding author on reasonable request.
